# Influence of a Polyherbal Mixture in Dairy Calves: Growth Performance and Gene Expression

**DOI:** 10.3389/fvets.2020.623710

**Published:** 2021-01-26

**Authors:** Cesar Díaz Galván, Estela Teresita Méndez Olvera, Daniel Martínez Gómez, Adrián Gloria Trujillo, Pedro Abel Hernández García, Enrique Espinosa Ayala, Monika Palacios Martínez, Alejandro Lara Bueno, Germán David Mendoza Martínez, Lucero Abigail Velázquez Cruz

**Affiliations:** ^1^Doctorado en Ciencias Agropecuarias, Universidad Autónoma Metropolitana Xochimilco, Ciudad de México, Mexico; ^2^Laboratorio de Biología Molecular, Departamento de Producción Agrícola y Animal, Universidad Autónoma Metropolitana Xochimilco, Ciudad de México, Mexico; ^3^Laboratorio de Microbiología Agropecuaria, Universidad Autónoma Metropolitana Xochimilco, Ciudad de México, Mexico; ^4^Departamento de Producción Agrícola y Animal, Universidad Autónoma Metropolitana Xochimilco, Ciudad de México, Mexico; ^5^Centro Universitario UAEM Amecameca, Universidad Autónoma del Estado de México, Amecameca, Mexico; ^6^Universidad Autónoma Chapingo, Texcoco, Mexico

**Keywords:** growth performance, Holstein calves, animal nutrition, nutrigenomics, feed plant additive

## Abstract

A polyherbal feed mixture containing (*Achyrantes aspera, Trachyspermum ammi, Citrullus colocynthis, Andrographis paniculata*, and *Azadirachta indica*) was evaluated in growing calves through blood chemistry, blood biometry, and gene expression during the pre-ruminant to weaning period. Forty Holstein calves (initial BW 45.6 ± 3.2 kg; 22.8 ± 0.9 days post birth) from a dairy farm were randomly assigned to the following treatments: 0, 3, 4, and 5 g/d of a polyherbal mixture, dosed in colloid gels with gelatin. Calves were housed in individual outdoor boxes with *ad libitum* access to a 21.5% CP calf starter and water and fed individually with a mixture of milk and a non-medicated milk replacer (22% CP). Blood samples were collected on day 59 for blood chemistry, blood biometry, and gene expression analysis in leukocyte through microarray assays. Immunoglobulins were quantified by enzyme-linked immunosorbent assay. The animals treated with the polyherbal mixture showed a quadratic effect on final body weight, daily weight gain, final hip height, and final thoracic girth. The best performance results were obtained with a treatment dose of 4 g/d. The serum IgG increased linearly with the treatment doses. Gene set enrichment analysis of upregulated genes revealed that the three biological processes with higher fold change were tight junction, mucin type O-Glycan biosynthesis, and intestinal immune network for IgA production. Also, these upregulated genes influenced arachidonic acid metabolism, and pantothenate and CoA biosynthesis. Gene ontology enrichment analysis indicated that the pathways enriched were PELP1 estrogen receptor interacting protein pathways, nuclear receptors in lipid metabolism and toxicity, tight junction, ECM-receptor interaction, thyroid hormone signaling pathways, vascular smooth muscle contraction, ribosome function, glutamatergic synapse pathway, focal adhesion, Hippo, calcium, and MAPK signaling pathways.

## Introduction

In dairy farms, respiratory and enteric diseases can occur, causing a high rate of mortality in young animals, significant economic losses (1, 2), and lower resistance to caloric stress (3, 4). In these animals, the feed intake is affected, which seriously compromises the development of the immune system (5). These diseases require the use of antibiotics which have been banned as growth promotors in several countries (6). There is an increase in public pressure for the usage of these drugs in animals, and therefore, the evaluation of feed plant additives with beneficial effects in adult ruminants on indicators of health and production are critical for raising calves (7–9).

In adult ruminants, a polyherbal mixture with phosphatidylcholine (PCho) and other nutraceutical metabolites results in improved performance, and health suggesting it may have beneficial effects on calves (10–12). Dietary PCho stimulates the IL-2 formation in the spleen and CD25, CD28, CD71 expression (13), improving the immune response. Choline is part of 1-alkyl-2-acetyl-sn-glycero-3-phosphocholine (platelet-activating factor), a recognized activator of immune response (14, 15), and rumen-protected choline can improve the immune response and health in cows (16–18); however, evaluations in calves before weaning are missing.

The objective of this study was to evaluate the effect of an herbal product, with PCho elaborated with medicinal plants from India (*Achyrantes aspera, Trachyspermum ammi, Citrullus colocynthis, Andrographis paniculata*, and *Azadirachta indica*), on the productive performance and immune response in Holstein calves. These plants have already been evaluated by our researching group (10–12) and the results obtained showed beneficial effects on production in adult animals. However, the activity of these plants on young animals is unknown.

## Materials and Methods

### Animals, Diet, and Experimental Design

The experimental procedures were performed in accordance with the recommendations of the CIOMS (19), observing the standards for ethics, biosafety, and animal well-being approved by the institutional committee. The experiment was conducted in a commercial farm located in the central part of the northern region of México (25°39' 14.4 “N 103°27' 27.8” W; elevation 1,139 m). The climate was semi-dry, with extreme temperatures, with an annual rainfall of 250.6 mm. Average temperature was 20.21°C, with a maximum of 33.60°C and a minimum of 5.59°C. Forty Holstein calves (initial BW 45.6 ± 3.2 kg) received individual doses of a polyherbal mixture (BioCholine, Indian Herbs). The doses were defined based on metabolic weight using results from sheep experiments (10, 12) and were as follows: 0, 3, 4, and 5 grams per day (g/d). The herbal products were prepared in colloid gels with gelatin to ensure individual daily consumption in the morning during the 90 days that the experiment lasted. The polyherbal-gelatin mixture (PGM) was prepared 1 day before administration. Calves were clinically healthy at the beginning of the experiment and had an age of 22.8 ± 0.9 days post birth. Calves were weighted and measured at the beginning and at the end of the experiment (90 days of evaluation). During weighing, the hip and wither heights as well as the thoracic girth were measured (20). All calves received colostrum following birth, and immunoglobulins in the blood were measured by refractometry.

Calves were individually placed in outdoor boxes (2.00 × 1.25 m), with water *ad libitum*, and fed twice a day (7.00 am and 17.00 pm). They received a mixture of milk (56%) and a non-medicated milk replacer (44% milk replacer Nu-3; Grupo Nu-3 balanced food, Guanajuato, México) with 4% moisture, 22% crude protein, 15% fat, 0.1% crude fiber, 6.0% ash, and 52.9% nitrogen-free extract (NFE), prepared with 130 g/L water. The milk powder (143 g/L) was reconstituted in hot water (65°C) and fed at a temperature of 39°C in a container; prior to feeding, it was mixed with milk at 130 g/L. Milk replacer was offered from day 4 of age twice a day (4 L each time) and was reduced to one meal on day 25 as starter intake was augmented. Form the second day of life, a commercial concentrate composed of sorghum grain, rolled corn, soybean meal, and cane molasses was offered from a bucket. The milk starter used was Premium Initiation Weaning Premature (Nuplen, Durango, México) with 13.0% moisture, 21.5% crude protein, 3.0% fat, 8.0% crude fiber, 7.0% ash, and 47.0% NFE. The starter concentrate was offered from the third day of age and was gradually increased.

### Samples and Data Collection

Daily intake of milk and starter concentrate was recorded. The variation in consumption between days was evaluated as an indicator of stability. Feed conversion was estimated as the ratio of kg of dry matter (DM) intake/kg of BW gain, including DM from starter, milk replacer, and milk starter. Calves were evaluated daily following the morning milk feeding between 06:00 and 10:00 to check for diarrhea, pneumonia, otitis, and other diseases. On day 59 of the experiment, pre-prandial blood samples of all animals were collected from the jugular vein using vacutainer tubes with sodium citrate, EDTA, and without anticoagulant. These blood samples were maintained under refrigeration (4°C) until use. Tubes without anticoagulant were centrifuged (Sigma 2-16 k, Germany) at 3,500 x g for 20 min to obtain blood serum, which was stored in Eppendorf tubes and kept in a freezer at −20°C until analysis. Cholesterol, glucose, total protein, albumin, and bilirubin were determined with an autoanalyzer Kontrolab 2017. The blood sample collected in disodium-EDTA was used for complete blood count (CBC). The CBC leukocyte differential count and the hematocrit were determined in the hematology analyzer QS Kontrolab EasyVet.

### Immunoglobulin Analyses

Calves were immunized against *Clostridium* spp. (Covexin 10 vaccine) on day 40 of the experiment, and serum obtained from the blood samples collected with sodium citrate was used to evaluate the antibodies against the antigen (*Clostridium* spp.). The antigen was bound to the microtiter plate and incubated overnight at room temperature. For antibody evaluation, 100 μL of the serum sample was added to each well and incubated (1 h, 37°C). Plates were decanted, and 100 μL of bovine IgG specific secondary antibody was added and incubated (1 h, 37°C). Then, 100 μL of TMB (3.3′, 5.5′-tetramethylbenzidine liquid) was added to each well, and the reaction was stopped with 100 μL of H_2_SO_4_ (1%). The plates were analyzed in an ELISA reader (Model 350, BioRad) at a wavelength of 540 nm.

### RNA Extraction From Blood Samples

Blood samples with sodium citrate from the control and the polyherbal treated group (4 g/d) were processed using SRL solution (Tris–HCl 10 mM pH 8, MgCl2 5 mM, and NaCl 10 mM) from a total volume of 24 mL of blood per experimental unit (calf). Total RNA was extracted from the leukocyte package using Trizol (Invitrogen). The RNA pellet was suspended in 20 μL of H_2_O DEPC 0.1% water and stored at −80°C until processing. RNA purity was determined by electrophoresis on a 1% agarose gel. The RNA samples were treated with DNases (Promega). The purified RNA was quantified by spectrometry using A_260_-A_280_ (21). Two pools of RNA (control and treatment) were prepared from eight biological repetitions (30 ug RNA) and stored at −70°C for 24 h and subsequently centrifuged for 15 min at 4°C.

### Microarray Analyses

Microarray analyses were carried out at the Unidad de Microarreglos del Instituto de Fisiología Celular (UNAM) in Mexico City, using a heterologous mouse M22K_10_16 chip to evaluate the differential expression of the genes. The arrangement evaluated 24,341 genes, and 10 μg of total RNA as synthesized to cDNA with the incorporation of nucleotides dUTP-Alexa555 and dUTP-Alexa647, using the CyScribe First-Strand cDNA Amersham labeling kit. Fluorophore incorporation was analyzed at an absorbance of 555 nm for Cy3 and 655 nm for Cy5. Equal amounts of cDNA were hybridized using the HybIT2 solution (TeleChem International INC). The arrangements were incubated for 14 h at 42°C, with three consecutive washes using 1 x SCC, 0.05% SDS at room temperature. We used the equipment and software ScanArray 4000 (Packard BioChips) for acquisition and quantification of the images of the arrangements. Microarray data analysis was conducted with the genArise software (http://www.ifc.unam.mx/genarise/). The list of genes considered upregulated (Up) and downregulated (Down) was analyzed with the bioinformatics tool DAVID [Functional Annotation Bioinformatics Microarray Analysis, (https://david.ncifcrf.gov/)], which allows the grouping of genes based on their functional similarity.

### Gene Ontology Analysis

Gene set enrichment analysis using gene ontology was applied to extract biological meaning from the identified differentially expressed transcripts; gene ontology terms with a *P*-value <0.05 were considered as enriched. The Database for Annotation, Visualization, and Integrated Discovery (DAVID) 6.8 Bioinformatic resource was used for gene set enrichment (22).

### Validation of Differential Genes Expressed Using Quantitative Real-Time PCR

First-strand cDNA was synthetized from 1,500 ng of total RNA using Oligo(dT) and RevertAid Reverse Transcriptase (Thermo Scientific). Specific primers for PPARβ and GAPDH were designed with Primer-BLAST (https://www.ncbi.nlm.nih.gov/tools/primer-blast/) (Table 1). Primers for PPARα and ACOXI were taken from previous studies (Table 1) (23). The qPCR was performed on a real-time PCR system Rotor-GeneQ (Qiagen), using Sybr Green for detection (Thermo Scientific). Amplification for PPARα and ACOXI was carried out for one cycle at 95°C for 10 min, followed by 45 cycles at 95°C for 15 s, 60°C for 60 s, and 70°C for 30 s. Amplification for PPARβ and Relb was carried out for one cycle at 95°C for 2 min, followed by 45 cycles at 95°C for 30 s, 57°C for 30 s, and 72°C for 30 s. Glyceraldehyde-3-phosphate dehydrogenase (GAPDH) was used as internal control for normalization. The 2^−ΔΔCt^ method was used to determine the relative mRNA quantification (24).

**Table 1:** Sequences (5′ to 3′) of the primers used in the qPCR.

	**Primers**		
**Gene**	**Forward**	**Reverse**	**pb**
PPARα	CAATGGAGATGGTGGACACA	TTGTAGGAAGTCTGCCGAGAG	95
ACOX	TCCTACTGTGACCTCCATCAA	GGGTCCCAAGTTCACGAATAG	143
PPARβ	GTGGCTTCTGTTCACCGACA	GAAGTGAGTGCTCTGGTCCC	257
GAPDH	GCCATCACCATCTTCCAGG	GGTAGTGAAGACCCCAGTGG	96

### Statistical Analyses

The Shapiro-Wilk test was used to test the normal distribution of variables. Data were analyzed using the R software (25), and orthogonal linear and quadratic polynomials were used to evaluate the effects of the polyherbal additive. The model used was: *Yij* = μ + τ*i* + *eij*, in which μ is the mean value, τ*i* is the treatment effect (fixed), and *eij* is the error term. The initial body weight was used as a covariate in daily gain and final BW (26). The number of cases of diseases and the doses of antibiotics were analyzed using the Kruskal Wallis test. If the quadratic effect was significant, the optimal concentration for daily gain was estimated using the first derived from the quadratic equation (27).

## Results

### Performance and Health Parameters in Calves

The animals treated with the polyherbal mixture showed a quadratic response in final BW, average daily gain (ADG), final hip height, and final thoracic girth, with the best numeric performance results with a dose of 4 g/d (Table 2). However, the optimal dose estimated by regression to maximum ADG was 2.47 ± 0.87 g/d of the polyherbal mixture. Starter intake was stimulated at a dose of 4 g (quadratic *P* < 0.001), whereas liquid ingestion showed a linear increase (*P* < 0.001), compensating for the reduction in solid consumption with no effect on feed conversion (Table 2). All calves received colostrum following birth, and immunoglobulins in the blood were measured by refractometry, the results obtained showed a concentration of 6.54± 0.525 g/dl. A reduction in otitis (quadratic effect *P* < 0.10) was observed with intermediate doses of polyherbal in the calves (Table 2), and a linear increment (*P* < 0.10) of anti-*Clostrdium* spp. IgG was detected by enzyme-linked immunosorbent assay.

**Table 2:** Effects of the polyherbal mixture treatment on growth performance and health status.

	**Polyherbal mixture (g/d)**				**SEM**	***P*****-value**	
	**0**	**3**	**4**	**5**		**Linear**	**Quadratic**
Average daily gain g/d	0.672	0.763	0.772	0.653	0.0206	0.89	0.008
Final BW kg	86.74	92.64	93.28	85.50	1.3904	0.78	0.008
Final wither height cm	90.06	91.18	91.33	90.51	0.5281	0.72	0.30
Final hip height cm	95.42	97.11	97.34	95.39	0.4268	0.53	0.05
Final thoracic girth cm	101.56	105.01	105.35	103.57	0.6006	0.20	0.02
Starter intake g/d	829	948	889	677	31.4816	0.02	0.001
Feed intake variation among individuals %	107.38	108.13	112.66	113.71	1.4026	0.12	0.96
Milk + replacer intake liters/d	2.454	2.564	2.582	2.666	0.0188	0.0001	0.63
Feed conversion ratio	2.15	2.06	1.94	2.07	0.0501	0.32	0.19
No. diarrheas	0.29	0.24	0.40	0.01	0.0595	0.81	0.70
No. pneumonias	3.61	0.50	3.20	0.88	0.5535	0.34	0.80
No. otitis	5.66	1.20	2.40	4.77	1.0023	0.79	0.06
Antibiotic doses	5.35	1.54	4.32	2.33	0.9383	0.41	0.60
Immunoglobulins (Anti-Clostridium IgG)	1.496	1.438	1.851	1.764	0.0766	0.07	0.62

*SEM, standard error of the mean*.

### Blood Chemistry and Biometry

Blood chemistry changes are shown in Table 3. The treatment with polyherbal mixture caused a reduction in glucose levels (linear *P* < 0.01), with a quadratic increase in B-OH butyrate (*P* < 0.05). Globulin levels were reduced (*P* < 0.10 quadratic) and albumin levels showed a quadratic increment (*P* < 0.05), resulting in a reduction in the albumin globulin ratio (quadratic *P* < 0.01). Urea showed a reduction (*P* < 0.10), whereas uric acid was not affected. The bilirubin level increased linearly (*P* < 0.01) with the polyherbal treatment; however, liver enzymes [alkaline phosphatase, ALP; lactate dehydrogenase, LDH; and aspartate aminotransferase, AST (GOT)] were not affected. Serum calcium and phosphorus remained unchanged. The results of the biometry are presented in Table 4. There was a reduction in neutrophils in band (*P* < 0.05). Lymphocytes, basophiles, and plasma protein were reduced with lower or intermediate doses of BioCholine (quadratic effect, *P* < 0.10).

**Table 3:** Effects of the polyherbal mixture on blood chemistry parameters.

	**Polyherbal mixture (g/d)**				**SEM**	***P*****-value**	
	**0**	**3**	**4**	**5**		**Linear**	**Quadratic**
Glucose mmol/L	4.101	2.975	3.480	2.621	2.5144	0.0001	0.47
B-OH Butyrate mmol	0.329	0.250	0.300	0.355	0.0156	0.35	0.03
Urea mmol/L	7.87	6.07	6.42	6.42	0.7066	0.08	0.09
Uric acid mmol/L	30.09	36.87	33.90	36.34	0.0276	0.24	0.49
Creatinine μmol/L	89.46	91.05	93.70	94.23	0.0309	0.60	0.94
Total protein mmol/L	69.9	69.2	70.8	70	0.1547	0.14	0.70
Globulin g/dL	3.04	2.76	2.76	2.90	0.0853	0.44	0.09
Albumin g/dL	3.95	4.16	4.32	3.99	0.1030	0.59	0.02
Ratio A/G	1.33	1.53	1.60	1.37	0.0563	0.59	0.01
Cholesterol mmol/L	24.26	22.91	22.85	23.72	2.7943	0.73	0.33
Bilirubin mmol/L	0.370	0.470	0.490	0.511	0.0211	0.01	0.34
ALP U/L	25.71	28.60	25.60	26.11	0.9695	0.72	0.98
LDH U/L	79.65	68.20	75.20	78.89	2.6029	0.85	0.21
AST (GOT) U/L	25.53	20.10	21.90	21.22	1.2873	0.45	0.49
Calcium mmol/L	2.30	2.22	2.52	2.29	0.2478	0.43	0.36
Phosphorus mmol/L	1.66	1.52	1.62	1.53	0.1352	0.32	0.69

*ALP, alkaline phosphatase; LDH, lactate dehydrogenase; AST (GOT), aspartate aminotransferase; SEM, standard error of the mean*.

**Table 4:** Effects of the polyherbal mixture on blood parameters.

	**Polyherbal mixture (g/d)**				**SEM**	***P*****-value**	
	**0**	**3**	**4**	**5**		**Linear**	**Quadratic**
Hematocrit %	35.13	34.7	35.8	36.05	0.335	0.37	0.72
Hemoglobin g/dl	11.91	11.7	12.15	12.08	0.116	0.48	0.82
Erythrocytes × 10^6^/ml	5.39	5.16	5.35	5.39	0.072	0.77	0.38
Mean corpuscular volume fl	65.73	67.29	66.82	66.86	0.576	0.58	0.54
Mean corpuscular Hemoglobin pg	22.27	22.71	22.68	22.4	0.209	0.85	0.41
Mean corpuscular hemoglobin concentration g/dl	33.9	33.72	33.94	33.51	0.111	0.25	0.53
Platelets × 10^3^/ml	407.22	376.20	449.1	458.22	13.912	0.09	0.52
Wintrobe sedimentation rate ml/h	0.27	0	0	0.55	0.150	0.58	0.24
Leucocytes × 10^3^/ml	11.4	11.57	12.4	11.05	0.391	0.96	0.49
Lymphocytes × 10^3^/ml	45.88	28.80	44.70	40.00	2.134	0.91	0.08
Monocytes × 10^3^/ml	4.55	3.90	5.00	4.66	0.253	0.56	0.77
Neutrophils segmented × 10^3^/ml	41.94	62.9	46.40	49.00	2.328	0.77	0.01
Neutrophils in band × 10^3^/ml	4.33	1.70	1.60	2.44	0.381	0.02	0.003
Eosinophils × 10^3^/ml	3.0	2.6	2.3	3.66	0.280	0.53	0.16
Basophiles × 10^3^/ml	0.27	0.10	0.00	0.22	0.055	0.59	0.09
Plasma proteins g/dL	9.09	8.64	8.98	9.42	0.115	0.22	0.07

*SEM, standard error of the mean*.

### Gene Expression Variations

The heterologous microarray represented ~22,000 transcripts, of which 2,442 were differentially expressed; a total of 1,093 and 1,349 transcripts were up- and downregulated, respectively. A total of 264 genes with values of +2.0 to 5.5 were upregulated and 401 genes with values of −2.0 to −5.8 were downregulated. The 30 most strongly up- and downregulated transcripts in blood leukocytes of weaning calves supplemented with polyherbal mixture are presented in Tables 5, 6. The most strongly upregulated transcripts include functional categories such as proto-oncogene (*Jun, Lck, Pdgfra*), tyrosine protein kinase (*Tyro3, Lck, Pdgfra*), immunoglobulin domain (*Tyro3, Lrn2, Pdgfra, Pdgfrl*), and ATP-binding (*Abcc8, Tyro3, Entpd2, Lck, Pdgfra, Plk1*) (Table 5). The most strongly downregulated transcripts include functional categories such as phosphoprotein (*Erc1, Rab11fip2, Rpusd2, Sh3d19, Tal1, Baz1a, Cobl, Cry2, Dapk1, Glycam1, Grhpr, Hmg20b, Kif2c, Lig1, Polr3d, Ptpn6, Sel1, Xirp2*), protein transport (*Erc1, Rab11fip2, Xpo5, Serp1*), and ubiquitin-like modifier conjugation (*Tal1, Cry2, Dapk1, Hmg20b, Kif2c, Polr3d*) (Table 6).

**Table 5:** Microarray analysis of differential gene expression profile in peripheral blood cells (upregulated genes).

**Gene symbol**	**Gene description**	**Fold change^a^**
Plk1	Polo-like kinase 1	5.5
Col11a1	Collagen, type XI, alpha 1	4.9
Tyro3	Protein tyrosine kinase 3	4.9
Klhdc9	Kelch domain containing 9	4.8
Casp2	Caspase 2	4.5
Entpd2	Ectonucleoside triphosphate diphosphohydrolase 2	4.2
Mcur1	Mitochondrial calcium uniporter regulator 1	4.1
Acoxl	Acyl-Coenzyme A oxidase-like	4.0
Lrrn2	Leucine rich repeat protein 2, neuronal	4.0
Snrpc	U1 small nuclear ribonucleoprotein C	3.9
Klra20	Killer cell lectin-like receptor subfamily A, member 20	3.9
Pdgfra	Platelet derived growth factor receptor, alpha polypeptide	3.9
Etd	Embryonic testis differentiation	3.8
Zfp27	Zinc finger protein 27	3.6
Prdx6	Peroxiredoxin 6	3.6
Abcc8	ATP-binding cassette, sub-family C (CFTR/MRP), member 8	3.6
Elovl2	Elongation of very long chain fatty acids (FEN1/Elo2, SUR4/Elo3, yeast)-like 2	3.6
Jun	Jun proto-oncogene	3.5
Rab3gap1	RAB3 GTPase activating protein subunit 1	3.5
Ccser2	Coiled-coil serine rich 2	3.5
Dsc1	Desmocollin 1	3.5
Mrps5	Mitochondrial ribosomal protein S5	3.4
Mga	MAX gene associated	3.4
TGF-β	transforming growth factor, beta 1	3.4
Defa14	Defensin, alpha, 14	3.3
Cenpp	Centromere protein P	3.3
Rpl41	Ribosomal protein L41	3.3
Pdgfrl	Platelet-derived growth factor receptor-like	3.3
Lck	Lymphocyte protein tyrosine kinase	3.2
Vpreb3	Pre-B lymphocyte gene 3	3.2

a *Polyherbal mixture treated vs. no treated animals*.

**Table 6:** Microarray analysis of differential gene expression profile in peripheral blood cells (downregulated genes).

**Gene symbol**	**Gene description**	**Fold change^a^**
Hmg20b	High mobility group 20B	−5.8
Cyp21a1	Cytochrome P450, family 21, subfamily a, polypeptide 1	−4.5
Ptgdr	Prostaglandin D receptor	−4.3
Serp1	Stress-associated endoplasmic reticulum protein 1	−4.3
Baz1a	Bromodomain adjacent to zinc finger domain 1A	−4.2
Cry2	Cryptochrome 2 (photolyase-like)	−4.2
Psmd8	Proteasome (prosome, macropain) 26S subunit, non-ATPase, 8	−4.2
Slc22a15	Solute carrier family 22 (organic anion/cation transporter), member 15	−4.2
Grhpr	Glyoxylate reductase/hydroxypyruvate reductase	−4.1
Kif2c	Kinesin family member 2C	−4.1
Rpusd2	RNA pseudouridylate synthase domain containing 2	−4.1
Cobl	Cordon-bleu WH2 repeat	−4.1
Glycam1	Glycosylation dependent cell adhesion molecule 1	−4.1
Xpo5	Exportin 5	−4.0
Dapk1	Death associated protein kinase 1	−4.0
Sel1l	Sel-1 suppressor of lin-12-like	−4.0
Xirp2	Xin actin-binding repeat containing 2	−4.0
Sh3d19	SH3 domain protein D19	−3.9
Lzic	Leucine zipper and CTNNBIP1 domain containing	−3.9
Rab11fip2	RAB11 family interacting protein 2 (class I)	−3.9
Erc1	ELKS/RAB6-interacting/CAST family member 1	−3.8
Lig1	Ligase I, DNA, ATP-dependent	−3.8
Wif1	Wnt inhibitory factor 1	−3.8
Tal1	T cell acute lymphocytic leukemia 1	−3.8
Gsc2	Goosecoid homebox 2	−3.8
Mc5r	Melanocortin 5 receptor	−3.8
Ptpn6	Protein tyrosine phosphatase, non-receptor type 6	−3.8
Polr3d	Polymerase (RNA) III (DNA directed) polypeptide D	−3.8
Grpr	Gastrin releasing peptide receptor	−3.8
G6pc3	Glucose 6 phosphatase, catalytic, 3	−3.8

a *Polyherbal mixture treated vs. no treated animals*.

Gene Set Enrichment Analysis (GSEA) of upregulated genes (Table 7) revealed that the three biological processes with higher fold change were tight junction (Fc: 3.7; *P* = 0.00002), mucin type O-Glycan biosynthesis (Fc: 3.7; *P* = 0.044), and intestinal immune network for IgA production (Fc: 3.4; *P* = 0.015). We observed reduced expression of genes related with tight junction (*Rab13, Src, Afdn, Cdc42, Llgl2, Mpp5, Myh10, Myh9, Myl9, Pard6g, Prkci Tjp1*); among these genes, there were four code for claudins (*Cldn-7, Cldn8, Cldn9, Cldn16*). The expression of *gcnt3* gene was reduced (Fc = −1.6); this gene is coded to the enzyme responsible for synthesis of all known β6 N-acetylglucosaminides. The treatment with polyherbal mixture reduced the expression of cytokines necessary to promote terminal differentiation and switching of immunoglobulin A class of B cells, explaining the downregulation of the genes involved in the intestinal immune network for IgA production (Cd40lg, Cd86, H2-DMb1, Il10, Madcam1, Tgfβ1, Tnfrsf13b). The microarray did not identify differentially expressed transcripts that codified for *Tnf-*α, *Ifn, Il-6*, and *Il8*, which code for inflammatory cytokines. In contrast, a reduced expression of *Tgf*β*1* (Fc: −3.4) and *Il-100* (Fc: −2.5) was shown.

**Table 7:** Gene ontology enrichment analysis in peripheral blood cells.

**GO biological process term**	**Count^a^**	***P*-Value**	**Fold change^b^**
Pantothenate and CoA biosynthesis	4	0.03	5.7
Tight junction	16	0.00002	3.7
Type II diabetes mellitus	7	0.031	3.6
Intestinal immune network for IgA production	7	0.015	3.4
Arachidonic acid metabolism	10	0.01	2.9
Neuroactive ligand-receptor interaction	23	0.019	1.7
Adherents junction	9	0.022	2.6
Ribosome	14	0.023	2.0
ECM-receptor interaction	10	0.025	2.3
Pathways in cancer	28	0.041	1.5
Focal adhesion	17	0.042	1.7
Mucin type O-Glycan biosynthesis	5	0.044	3.7
Endocytosis	20	0.047	1.6

a *Number of genes associated*.

b *Polyherbal mixture treated vs. no treated animals*.

Analysis of gene enrichment with positive regulation identified pantothenate and CoA biosynthesis (*P* = 0.03), arachidonic acid metabolism (*P* = 0.01), and diabetes mellitus type 2 (*P* = 0.01) (Table 7). The treatment with polyherbal mixture increased the expression of four genes whose transcription is related to pantothenate and CoA biosynthesis (*Enpp1, Enpp3, Pank4*, and *Vnn3*).

**Figure 1:**
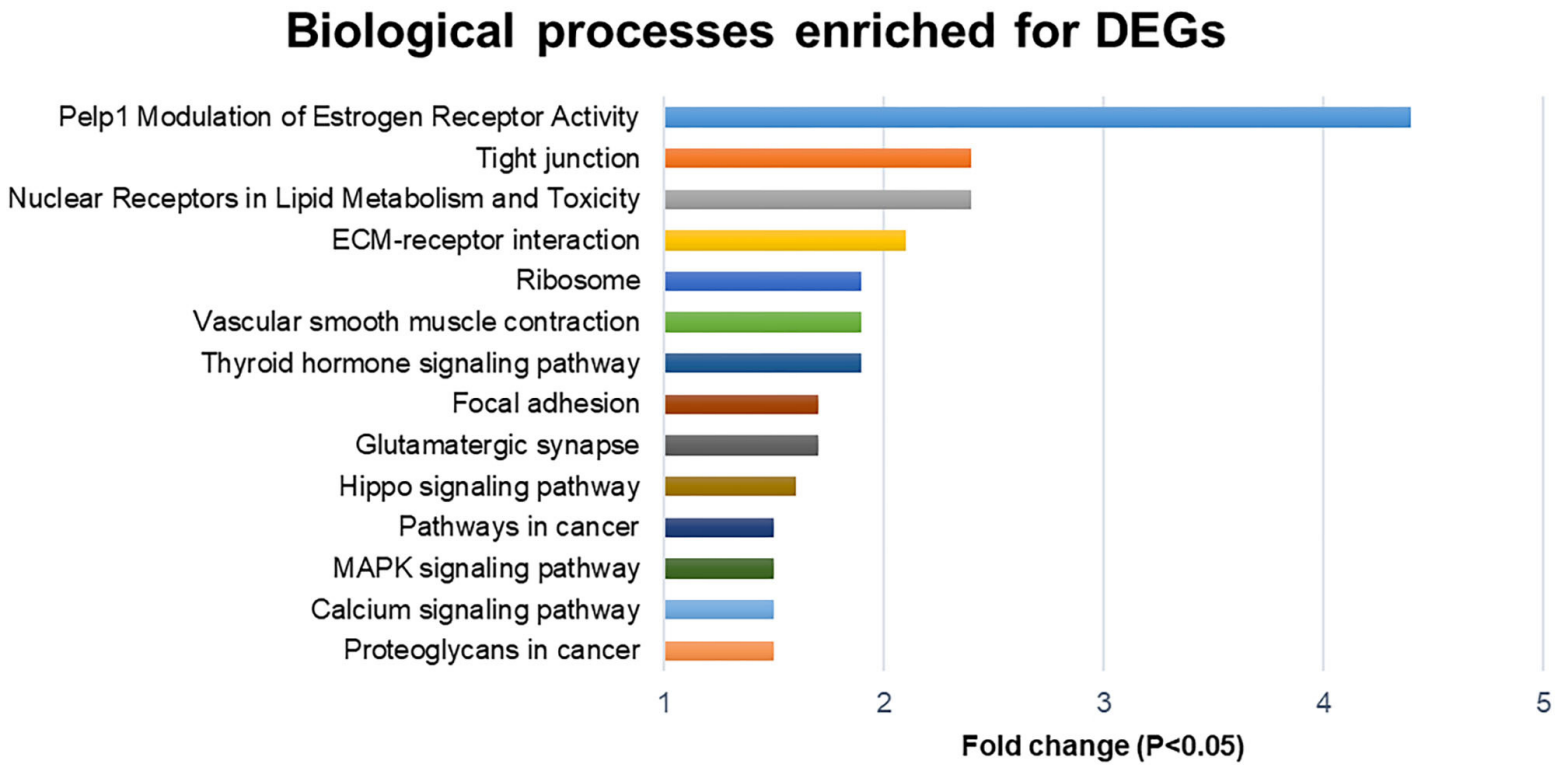
Biological processes enriched for differentially expressed genes (DEGs; down and upregulated transcripts).

Analysis of total up and downregulated transcripts (Figure 1), indicates an important modulator effect of polyherbal mixture treatment on *Pelp1* modulation of estrogen receptor activity (Fold change, Fc = 4.4; *Src, Esr1*, and *Mapk1*), nuclear receptors in lipid metabolism and toxicity (Fc = 2.4; *Abca1, Abcb1b, Cyp1a2, Cyp27b1, Nr1h3, Nr1i3, PPARa, PPARd, Rara*, and *Rarg*), and tight junction (Fc: 2.4).

Analysis of gene expression by qPCR (Table 8) indicates that PPARα and PPARβ gene transcription were upregulated (*P* = 0.15) and downregulated (*P* = 0.05), respectively.

**Table 8:** Analysis of gene expression (^ΔΔ^CT).

	**Polyherbal mixture (g/d)**						
**Gene**	**0**	**3**	**4**	**5**	**SEM**	**Linear**	**Quadratic**
PPARα	1.24	21.57	14.84	42.49	6.35	0.53	0.15
PPARβ	3.33	2.99	0.82	0.81	0.59	0.84	0.05

*PPARα, peroxisome proliferator activated receptor α; PPARβ, peroxisome proliferator activated receptor β/δ; SEM, standard error of mean*.

## Discussion

### Calf Parameters

The polyherbal mixture provides conjugates of choline (mainly PCho) and secondary metabolites that, at low doses, have nutraceutical properties (11), which explains the improved response in growth (quadratic effect). Growing calves have a high protein synthesis, and choline participates as a methyl donor (via betaine) necessary for the methylation of DNA, RNA, and proteins (28, 29), in addition to the synthesis of the phospholipids of cell membranes and plasma lipoproteins (30) and to multiple functions in the organism (31). Few choline evaluations have been reported in calves since deficiencies of this nutrient were reported in the 1950s (29). Although it is included in milk replacers and starters, as suggested by the NRC (32), it is not supplemented after weaning. However, studies with growing lambs show a quadratic response to supplementation in the form of rumen-protected choline (RPC) or with the polyherbal mixture evaluated here (11, 12, 30). The positive response to choline is due to the rapid growth rate of Holstein cattle after weaning, and genetic selection can lead to increased requirements for this nutrient. Some RPC assessments have been made in steers in growing and finishing stages. In these studies, it was shown that when the animals approach to mature weights, the response to the nutrient is lower. Response to RPC was also reduced as the animal approaches mature weight (33–35).

In calves, few phytogenic feed additives with essential oils have been evaluated (36), which report higher intake of starter and better feed efficiencies and gain, whereas other authors did not find intake responses (37, 38). It is not clear if the changes in consumption showed in this investigation were due to some secondary metabolites of polyherbal mixture or by the conjugates of choline. In lambs that responded to the same polyherbal mixture in ADG, intake was not affected (12). However, in an experiment with graded levels of RPC, intake showed a quadratic response parallel to the gain response (39). Changes in thoracic girth in intermediate doses indicate greater rumen development, but there are few reports where nutrients in the diet affected these variables, and generally, those changes are not noticeable (40–42).

Regarding the evaluated health parameters, the reduction in otitis can be explained by some antibacterial metabolites in the polyherbal mixture (11), by the choline antioxidant capacity (43) that may play a preventive role in otitis (44), or by improving the immune response (45); this is important as in calves, otitis is commonly linked to pneumonia (46). However, more studies are necessary to confirm these results. The polyherbal mixture improved the immune response to vaccination, as observed in the quantifications of serum immunoglobulins in some feed plant additives (8), and could be related with the upregulation of some genes such as *Tyro3, Lrn2, Pdgfra*, and *Pdgfrl*.

### Blood Chemistry and Biometry

The blood chemistry values were in the normal range for calves (47, 48). Only glucose showed a slight reduction in animals supplemented with herbal choline. The polyherbal mixture contains *Azadirachta indica*, which has shown hypoglycemic effects (49). However, these effects do not affect leukocytes, which was indicated by the downregulation of the expression (−3.8 fold lower than in the control group) of the *g6pc3* gene, which codes for glucose-6-phosphatase involved in the homeostatic regulation of glucose in monocytes and neutrophils. The major function of glucose 6-phosphatase-β is to provide recycled glucose to the cytoplasm of neutrophils to maintain normal function (50).

The change in butyrate may be an indicator of ruminal development. Glucose is reduced because as calves are growing, they depend more on volatile fatty acids. Serum calcium and phosphorus also indicate that the feed additive did not affect the mineral balance of these elements, regardless of differences in feed intake of starter (51, 52). The calves were healthy, and minor changes in some metabolites were detected, which were, however, not associated with infectious agents or chronic inflammatory processes. Urea blood reductions indicate an adequate general situation of protein metabolism (53), confirmed by uric acid, a metabolic product of protein metabolism; the creatinine levels indicate that the kidney function was not affected (54).

The main serum indicators used to evaluate liver alterations include AST(GOT), ALT, bilirubin, total protein, and total albumin (55) and were not altered by the treatment with polyherbal mixture, confirming that the liver was not damaged. Changes in bilirubin levels can be explained by the reduction in the expression of *ugt2b37* (UDP-glucuronosyltransferase), which codes for the enzyme involved in bilirubin metabolism (56) and catalyzes the transformation of unconjugated or indirect bilirubin into conjugated or direct bilirubin by the addition of glucuronic acid. Some herbal supplements and extracts with polyphenolic compounds can have inhibitory effects on the gene responsible for glucoronidation of bilirubin (57). Since the calves in our study were healthy, presumably cross-reactions from some metabolites from herbal choline could be responsible for the increase in bilirubin, rather than liver injury. According to a previous study, serum bilirubin concentration is not considered as a sensitive indicator of liver disease in young calves (55).

In other experiments with feed plant additives, serum protein levels were not affected (36), and the results may vary depending on the mechanism of action of phytobiotics, secondary metabolites, and doses. Liver enzymes as ALT is the most specific test to detect liver dysfunction as well as LDH (55), suggesting that the polyherbal mixture evaluated does not have hepatotoxic metabolites and its choline conjugates could have an antioxidant effect (58) and other beneficial effects for liver function of this nutrient (59). The Polyherbal mixture has several chemical compounds, and some of them have already been reported to possess bactericidal properties (11, 12). Therefore, this could affect the structure and composition of the microbiota, affecting the ruminal fermentation. These could explain the lack of correlation between the starter intake and blood B-OH butyrate.

The biometry results indicate that calves were healthy and within the reported values (60, 61), similar to those shown for 1-month-old calves (62). The reduction in neutrophils in band was not correlated with the presence of bacterial disease (63). In fact, the polyherbal mixture treatment stimulated genes such as *Defa14*, recognized as antimicrobial peptides (64). In one experiment, aflatoxin B1 decreased neutrophils in calves, and the administration of choline helped to reduce the toxic adverse effects (65).

### Gene Expression Variations

Microarray analysis allowed us to detect significant changes in gene expression induced by metabolites in the polyherbal mixture. The analysis of changes in gene expression by bioinformatics analysis (GSEA) resulted in the identification of specific metabolic processes involved in calf metabolism. Although this work only evaluates gene expression in leukocytes, the variations in the expression observed in these cells indicates variations produced in other cells.

The changes in gene expression could explain some variations observed in calf performance and changes in blood variables. The possible physiological implication of this variation raises important questions about the plant metabolites. For example, the reduction in the expression of *gcnt3* gene and its implications may be important since this gene coded the enzyme responsible for the synthesis of all β6 N-acetylglucosaminides, which play an important role in O-linked glycosylation in mucin biosynthesis and, consequently, has implications on the functional state of the mucosa of the intestinal and respiratory tracts (66).

In calves treated with polyherbal mixture, the expression of Claudin genes in leukocytes was reduced. Claudins are the main transmembrane proteins that regulate the intestinal epithelial permeability, controlling ion and microbial product diffusion through the epithelial cell layer (67, 68). Malmuthuge et al. (69) have reported that the diet regulates the expression of Claudin 4 and occludin, altering the absorption of macromolecules. Considering that modification in expression of these genes was induced by metabolites in the polyherbal mixture, the same response could be present in cells of the gastrointestinal tract, thereby modifying intestinal permeability and the absorption of macromolecules.

The genetic enrichment analysis provided a perspective on the changes induced by the polyherbal treatment at different metabolic stages. Increased pantothenate and CoA biosynthesis (Table 7) has important consequences on energy metabolism, which were manifested in calf growth, and expression of *Pank4*, which encodes pantothenate kinase, a regulatory enzyme in CoA biosynthesis. The gene *Pank4* phosphorylates the vitamin pantothenate to 4′-phosphopantothenate, which is converted into CoA and participates in the tricarboxylic acid cycle, fatty acid metabolism, and numerous other reactions of intermediary metabolism (70). The *Pank4* mRNA is upregulated at high glucose concentrations to allow higher flux through the tricarboxylic acid cycle (71).

Coenzyme A biosynthesis and the increased PPARα gene expression induced by choline have important implications. Here, PPARα stands out because the group of nuclear proteins called PPAR (peroxisome proliferator-activated receptor) plays a key role in the regulation of metabolic pathways such as lipid metabolism (72), and it is possible that the over-expression of the genes *Acoxl* (acetyl CoA-oxidase) and the gene *Cpt2* (carnitine palmitoyl transferase 2) is mediated by the positive regulation of PPARα and by other effects of choline, such as antioxidant effects, lipotropic effects, or through the activity of *GSH-Px* (43).

The treatment with polyherbal mixture also stimulated a group of genes associated with nuclear receptors in lipid metabolism and toxicity (Figure 1). There are PPAR receptors for fatty acids and *cytochrome P450* genes involved in lipid metabolism; some genes of *cytochrome P450* are related to the detoxification of environmental toxins and the metabolism of xenobiotics and drugs (73); this explains the beneficial effects of choline in calves with aflatoxins (65). The ECM-receptor interaction pathway is involved in adipose tissues and important for adipogenesis and functional macromolecules (glycosaminoglycans, collagen, elastin, fibronection, and lammin), but there are complex interactions with other cells, which need to be addressed (74).

Ribosomal gene stimulation suggests that polyherbal treatment simulates protein synthesis, which coincides with the growth response observed in calves; the ribosomes are the cellular organelles responsible for protein synthesis in cells (75). The effects of glutamatergic synapse pathway stimulation in calves are unclear, but it is known that glutamate is an important neurotransmitter in the central nervous system and that its stimulation affects receptors, channels, and neurotransmitter transporters (76), with potential effects on the stress response. In their review, Musazzi et al. (77) present evidence that glucocorticoids and acute stress increase extracellular glutamate levels, affecting glutamate release in the brain; the authors highlight that chronic stress may reduce glutamate metabolism. In this sense, animals in confinement can be chronically stressed, and calves receiving a dose of 3 g/d showed better health and improved weight gain. This leads us to infer that this route could have reduced stress.

Focal adhesion processes may have improved the health conditions of calves since they participated in cell migration and invasion (78). Liu et al. (79) have demonstrated that the phytoestrogen genistein at high concentrations reduced the damage in estradiol-induced cardiovascular cells, affecting the focal adhesion pathway. The Hippo pathway plays a role in organ size and morphogenesis control as well as cancer development (80). Growth is an important parameter in calves, and changes in morphometric measurements were observed in this study.

The overall effects reflect the combined effects of conjugates of choline and secondary metabolites from polyherbal mixture (11) on the metabolism of lipids and glucose (59, 81) and on the immune response (13, 17, 18); this is in agreement with the demonstrated beneficial effects of natural products from traditionally used medicinal plants (72), and it can have been relative compared with the gene expression in experiments with synthetic choline products (82).

The treatment with polyherbal mixture could have an important effect in PPARα gene expression. It is important for immunity in calves because it is predominantly expressed in T and B cells (83). Also, PPARα is an important regulator of gene expression in rumen epithelium during the period from pre-ruminant to ruminant and can be involved in the mediation of energy metabolism within the rumen epithelium to support rumen development and differentiation of the ruminal papillae during weaning (84, 85). Metabolically, PPAR receptors induce the proliferation of peroxisomes in cells, a process that generates the transcription of the acyl-CoA oxidase (*AcoxI*) gene (86); however, *AcoxI* gene validation by qPCR did not confirm the microarray results where *AcoxI* was upregulated. On the other hand, the increased PPARα gene transcription (Table 8) can be explained by the mechanism of action of choline conjugates, mainly phosphatidylcholine, which we assume is the main metabolite involved in the stimulation of gene expression of *Pla2g6, Pla2g4e*, and *Pla2g2d*, which encode phospholipases A2, involved in the hydrolysis of arachidonic acid in the C2 position of phosphatidylcholine (87). The release of arachidonic acid stimulates the expression of the *Ptgds* and *Ptgis* genes, which encode enzymes involved in the cyclooxygenase pathway for the production of prostaglandins and prostacyclines. The genes *Alox12* and *Alox15* encode enzymes involved in the lipoxygenase pathway, synthesizing the acids 12-HPETE (12-hydroperoxyeicosatetraenoic acid), 15-HPETE (15-hydroperoxyeicosatetraenoic acid), and Cyp4a10, which encode monooxygenase to synthesize 20-hydroxyeicosatetraenoic acid (20-HETE). These fatty acids derived from arachidonic acid are incorporated into the β-oxidation process, but they also act as agonists of the expression of the PPARα gene (88), a transcription factor that reduces the inflammatory response by sub-regulation of genes induced by cytokines, an effect attributed to the direct interaction of PPARα with the p65 subunit of *NF-kB*, reducing its link with DNA (89). This would explain the reduction in the expression of *Rel* and *RelA* genes of *NF-kB*. The absence of differentially expressed genes (*Tnf*α, *Ifn, Il2, Il6, Il8*) that code for proinflammatory cytokines and the reduced production of profibrotic factors such as *Tgf-*β*1* (Table 5) would also explain the reduction in the expression of anti-inflammatory interleukin 10 gene in calves (90).

In addition, the activation of the transcription factor PPARα by arachidonic acid or other metabolites derived from arachidonic acid, such as 20-HETE, and the binding with its receptor cis-9 retinoic acid reduced the expression of the *Scd1, Nr1h3, Gyk2*, and *Aqp7* genes, involved in gluconeogenesis, lipogenesis, and cholesterol metabolism, and increased the expression of genes *Acsl4, Cyp4a1, Acoxl, Cpt2*, and *Mmp10*, involved in fatty acid transport and oxidation and in adipocyte differentiation (90, 91).

In contrast to PPARα, the downregulation of PPARβ gene transcription is explained for a specific agonist. All PPAR receptors have a ligand-binding domain (LBD), enabling PPAR activation by polar structure ligands, particularly by fatty acids (91). Arachidonic acid (AA) and AA metabolites connect with LBD and act as natural activators of PPARα (92). However, Naruhn et al. (93) identified that 15-HETE strongly induces the expression of PPARβ and, conversely, inhibition of 15-HETE synthesis reduces PPARβ transcriptional activity. In this sense, upregulated genes encode for lipoxygenases that synthetize 12-HPETE and 15-HPETE (arachidonate 12-lipoxygenase and arachidonate 15-lipoxygenase), but microarray did not identify differentially expressed genes that encoded for glutathione peroxidase (Gpx), responsible for the synthesis of 12-HETE and 15-HETE from 12-HPETE and 15-HPETE.

In weaning calves, stress triggers inflammatory responses (94). Prolonged stress suppresses immune functions to enable survival, consequently increasing disease susceptibility (95). Lewis et al. (45) have shown that phosphatidylcholine can stimulate the immune response, resulting in up-regulated PPARα gene activation, modulating the pro-inflammatory response and preventing its excessive activation (91). Optimizing calf health from birth to weaning will impact long-term health and animal welfare (96), allowing the animals to perform better in their first calving and lactation (97).

## Conclusions

The polyherbal mixture treatment, at doses of 4 g/d, could improve growth and health status during the pre-ruminant to the weaning period through modification of gene expression. Gene expression analysis confirmed that polyherbal treatment could improve the metabolism of lipids, carbohydrates, proteins, and also immune response. However, more studies are necessary to provide evidence of this aspect. Our results confirm the usefulness of plant compounds in animal feed.

## Data Availability Statement

The raw data supporting the conclusions of this article will be made available by the authors, without undue reservation.

## Ethics Statement

The animal study was reviewed and approved by the Academic Committee of Doctorado en Ciencias Agropecuarias at Universidad Autónoma Metropolitana- Xochimilco, Mexico City, Mexico.

## Author Contributions

CD and AG performed the experiments and wrote section of the manuscript. EM and DM contributed to conception of the study, participated in its design, and helped to draft the manuscript. GM and PH participated in the design of the experiments and performed the statistical analysis. MP carried out the immunoassays. EE and AL participated in the coordination of the fieldwork. LV carried out the animal care. All authors contributed to manuscript revision, read, and approved the submitted version.

## Conflict of Interest

The authors declare that the research was conducted in the absence of any commercial or financial relationships that could be construed as a potential conflict of interest.
